# Considerations for selecting immunodeficient mouse strains for cancer xenograft models

**DOI:** 10.1186/s42826-026-00292-8

**Published:** 2026-08-25

**Authors:** Jiwon Lee, Joohee Jung

**Affiliations:** College of Pharmacy, Duksung Women’s University, Seoul, Republic of Korea

**Keywords:** Xenograft model, Immunodeficient mouse, Cancer research

## Abstract

Preclinical (in vivo) experiments for efficacy and toxicity are predominantly conducted in mice. Nevertheless, human-derived cells, tissues, and organoids are generally rejected by the immune system of mice. Thus, immunodeficient mice are used as xenograft models to overcome immune rejection in cancer research. Xenograft mouse models are divided into ectopic, orthotopic, and metastatic models based on the transplant sites. Xenograft mouse models are established by transplanting cancer cells, tissues, or organoids derived from patients. These models show diverse success rates and production times depending on the transplant materials and recipient mice. In this review, mouse strains for the establishment of xenograft models in cancer research are introduced. Genetic engineering of immune cells, such as T cells, B cells, and natural killer cells, shows specific properties. To select an immunodeficient mouse strain, the differences in the characteristics of the models must be understood. Optimized mouse models can provide information to investigate cancer progression and evaluate anticancer drugs.

## Background

Cancer remains one of the leading causes of death worldwide and metastatic disease is responsible for the vast majority of cancer-related mortality [1]. The development of effective anticancer therapies requires animal models that can reproduce tumor growth, invasion, metastasis, and treatment responses in vivo. Among laboratory animals, mice are the most widely used because of their small size, short reproductive cycle, and extensive genetic characterization [2, 3]. Unlike in vitro systems, mouse models allow investigation of tumor-host interactions, angiogenesis, immune responses, and metastatic dissemination [4]. Early cancer models relied on transplantation of murine tumors into syngeneic mice. Although useful, these systems do not accurately reflect human cancer biology. The major obstacle to transplantation of human tumors into mice is immune rejection. The discovery of naturally occurring mutations affecting lymphocyte development led to the creation of immunodeficient mouse strains, including nude and severe combined immunodeficient (SCID) mice. Subsequent generations of immunodeficient strains incorporated defects in T cells, B cells, natural killer (NK) cells, macrophages, and dendritic cells, enabling more efficient engraftment of human tissues [5, 6]. These advances established the foundation for modern xenograft models and, more recently, humanized mouse systems used in immuno-oncology research [7]. Because each strain differs in immune status, engraftment efficiency, metastatic potential, and experimental applicability, appropriate strain selection is critical [8–10]. This review summarizes the characteristics, strengths, and limitations of commonly used immunodeficient and humanized mice and provides practical guidance for selecting suitable xenograft models.

### Brief overview of cancer xenograft models

Cancer xenograft models are designed to evaluate tumor biology and therapeutic responses using human-derived materials. The most established system is the cancer cell line-derived xenograft (CDX) model, generated by implanting cultured human cancer cell lines into immunodeficient mice. CDX models are inexpensive, highly reproducible, and suitable for large-scale drug screening. However, prolonged in vitro culture can alter genomic characteristics and reduce tumor heterogeneity [11]. Consequently, these models often fail to reproduce the complexity of patient tumors and their microenvironment. To overcome these limitations, patient-derived xenograft (PDX) models were developed [12]. PDX models are established by directly implanting freshly obtained patient tumor tissues into immunodeficient recipient mice. Compared with CDX models, PDX tumors retain histological architecture, molecular characteristics, genetic diversity, and intratumoral heterogeneity. Drug responses observed in PDX models frequently resemble those of the original patients, making them valuable tools for translational research and precision medicine [13]. Nevertheless, PDX establishment can be time-consuming, engraftment rates vary considerably among tumor types, and human stromal components are progressively replaced by murine stroma during serial passage. Patient-derived organoid xenograft (PDOX) models represent another advancement [14]. In this approach, organoids generated from patient tumors are transplanted into mice. PDOX models preserve key genetic and pathological features while offering greater flexibility and scalability than conventional PDX systems.

Xenografts can also be categorized according to transplantation site. Ectopic models are typically established by subcutaneous implantation into the flank or hind limb and allow convenient monitoring of tumor growth [13]. Orthotopic models involve implantation into the corresponding anatomical organ, thereby reproducing local microenvironmental conditions more accurately. Although technically demanding, orthotopic models often better reflect tumor progression and metastatic behavior observed in patients.

Metastasis models may be classified as experimental or spontaneous. Experimental metastasis is usually induced by intravenous injection of tumor cells through the tail vein. This approach is simple and reproducible but bypasses early metastatic events. In contrast, spontaneous metastasis models allow dissemination from a primary tumor site and therefore more closely mimic clinical disease [15]. However, successful establishment depends on both the tumor type and recipient strain.

### Immunodeficient mouse strain selection

#### Balb/c-nu (Nude) mouse

Nude mice possess a spontaneous mutation in the Foxn1 gene that results in athymia and absence of functional T lymphocytes [16, 17]. Their characteristic hairless phenotype facilitates imaging applications and experimental handling. Although adaptive immunity is impaired, innate immune functions, including NK-cell activity, remain relatively intact [18]. Consequently, engraftment rates are lower than those observed in more severely immunodeficient strains.

Despite this limitation, nude mice remain widely used in oncology research because they are economical and easy to maintain. They are particularly suitable for routine CDX studies and evaluation of tumor growth. In some fields, including thyroid cancer research, nude mice remain preferred because their biological environment appears favorable for tumor progression compared with other immunodeficient strains [19, 20].

#### Severe combined immunodeficient (SCID) mouse

SCID mice carry a spontaneous mutation of *protein kinase*,* DNA-activated*,* catalytic subunit* (*Prkdc*) in CB-17/Icr mice, resulting in defective V(D)J recombination and absence of functional T and B cells [21, 22]. Innate immune populations, including macrophages, granulocytes, and NK cells, are still present [23]. Compared with nude mice, SCID mice exhibit improved acceptance of human tissues. However, SCID mice possess several drawbacks. Residual innate immunity may still limit engraftment, and some animals develop age-related immune leakage characterized by partial recovery of lymphocyte function [24, 25]. Furthermore, because *Prkdc* is involved in DNA repair, SCID mice display increased sensitivity to radiation, limiting their utility in studies involving irradiation.

#### Non-obese diabetic (NOD)-SCID mouse

NOD-SCID mice combine the SCID mutation with the non-obese diabetic (NOD) genetic background. In addition to deficiencies in T and B cells, these mice exhibit impaired macrophage function, reduced complement activity, decreased NK-cell activity, and lower dendritic-cell function. These characteristics substantially improve acceptance of human cells and tissues [5, 6, 8–10].

The NOD background contains a polymorphism affecting SIRPα signaling, which reduces macrophage-mediated elimination of human cells [26–28]. Consequently, NOD-SCID mice generally demonstrate higher engraftment rates than nude or SCID mice. Nevertheless, immune leakage can still occur in older animals, and careful consideration of animal age is required.

#### NOD.Cg-*Prkdc*^*scid*^*Il2rg*^*tm1Sug*^/Jic (NOD/Shi-*scid*, IL-2Rγ^null^, NOG) mouse

NOG mice were generated by introducing IL-2 receptor γ (Il2rg) chain deficiency into the NOD-SCID background [29]. Loss of IL2rg chain signaling disrupts multiple cytokine pathways required for lymphoid development, producing severe defects in T cells, B cells, and NK cells [30]. Macrophage, dendritic-cell, and complement functions are also markedly impaired [29, 30]. A major advantage of NOG mice is the stability of their immunodeficient phenotype. Unlike SCID-based strains that may exhibit age-dependent immune leakage, NOG mice maintain profound immunodeficiency throughout life [31]. This property makes them highly suitable for PDX establishment, stem-cell transplantation, and humanization procedures.

#### NOD.Cg-*Prkdc*^*scid*^*Il2rg*^*tm1Wjl*^/SzJ (NOD/LtSz-*scid*, IL-2rγ^null^, NSG) mouse

NSG mice share many characteristics with NOG mice but possess a complete null mutation of the *Il2rg* gene [32, 33]. They lack T cells, B cells, and NK cells and display extensive impairment of innate immune functions [33]. These features make NSG mice among the most permissive recipients currently available for human cell transplantation [33]. NSG mice frequently demonstrate superior engraftment efficiency and can support growth of very small numbers of implanted tumor cells [34, 35]. They are particularly useful for studies involving primary patient samples, cancer stem cells, and spontaneous metastasis. Importantly, metastatic patterns observed in NSG mice often resemble those seen in human patients, enhancing their translational relevance [36].

#### NOD.Cg-Rag1^tm1Mom^ Il2rg^tm1Wjl^/SzJ, NOD.Cg-Rag2^tm1Fwa^ Il2rg^tm1Sug^/Rj (NRG) mouse

NRG mice were developed using the NOD background together with a *Rag1* or *Rag2* gene deficiency and *Il2rg* disruption [37]. Similar to NOG and NSG mice, they lack functional T cells, B cells, and NK cells and exhibit profound defects in innate immunity [38]. The principal distinction is that Rag deficiency does not impair DNA repair in non-lymphoid tissues [38]. Consequently, NRG mice tolerate irradiation more effectively than SCID-based strains [39]. This advantage makes them attractive for experiments involving radiation conditioning, hematopoietic stem-cell transplantation, or DNA-damaging therapies [40].

#### Humanized mouse

Although conventional immunodeficient mice support growth of human tumors, they lack a functional human immune system. Humanized mice were developed to overcome this limitation and enable investigation of interactions between tumors and human immune cells [41–43].

The humanized-peripheral blood mononuclear cells (Hu-PBMC) model is generated by intravenous injection of human PBMC. Human T cells expand rapidly, allowing efficient evaluation of immune checkpoint inhibitors, bispecific antibodies, and chimeric antigen receptor (CAR)-T therapies [44]. However, xenogeneic graft-versus-host disease (GvHD) develops relatively quickly, restricting experimental duration [45].

The humanized-hematopoietic stem cell (Hu-HSC) model is established by transplantation of human CD34-positive hematopoietic stem cells into irradiated immunodeficient recipients, typically NSG or NOG mice [44]. This approach supports multilineage immune reconstitution, including T cells, B cells, NK cells, and myeloid populations. Hu-HSC mice are therefore useful for long-term studies of tumor immunity and immunotherapeutic responses [44]. Nevertheless, establishment requires several months, substantial resources, and specialized expertise [45].

The humanized-bone marrow-liver-thymus (Hu-BLT) model is considered the most sophisticated humanized platform. It is generated by implantation of human fetal liver and thymic tissues combined with autologous hematopoietic stem cells. Because T-cell development occurs within a human thymic environment, antigen-specific immune responses are more physiologically relevant than in other humanized systems. However, ethical considerations, technical complexity, high costs, and risk of graft-versus-host disease limit widespread use [46, 47].

To establish humanized mice, NOG, NSG, and NRG mice were used as recipients [43, 48]. The humanized mouse strains have been developed to study immuno-oncology [49]. Humanized mice are appropriate for understanding the tumor microenvironment [47] and evaluating immunotherapeutic agents in cancer [50].

### Integrated framework for selecting mouse strains in xenograft models

#### Key principles of strain selection

Selection of an appropriate mouse strain should be guided primarily by the degree of immunodeficiency required for successful engraftment. In general, engraftment efficiency increases progressively from nude to SCID, NOD-SCID, and then NOG, NSG, or NRG mice (Table 1). Deeper immunosuppression usually improves transplantation success (Table 2) but also increases experimental cost and housing requirements. The intended biological question is equally important. Studies focusing solely on primary tumor growth may not require the most severely immunodeficient strains, whereas investigations of metastasis, stem-cell biology, or patient-derived tissues often benefit from highly permissive recipients. Differences in tumor-host interactions can substantially influence outcomes and should therefore be considered during experimental planning [51].

**Table 1:** Immune system differences between mouse strains

Mouse strain	Immune system					
	T cell	B cell	NK cell	Complement activity	Macrophage, Dendritic cell	IL-2 receptor γ chain
Nude	Absent	Present	Present	Normal	Present	Present
SCID	Absent	Absent	Present	Normal	Present	Present
NOD-SCID	Absent	Absent	Reduced	Defective	Impaired	Present
NOG	Absent	Absent	Absent	Defective	Impaired	Absent
NSG	Absent	Absent	Absent	Defective	Impaired	Absent
NRG	Absent	Absent	Absent	Defective	Impaired	Absent

**Table 2:** Characteristics of mouse strains

Mouse strain	Genetic characteristics	Tumor engraftment	Metastasis	Ref.
Nude	*Foxn1*	Capable	Rarely	[52–54]
SCID	*Prkdc* ^*scid*^	Capable	Rarely, Long time	[55]
NOD-SCID	*NOD background (Sirpa*^*Nod*^*)*,* Prkdc*^*scid*^*mutation*	Increased	Malignant	[55, 56]
NOG	*NOD background (Sirpa*^*Nod*^*)*,* Prkdc*^*scid*^*mutation*, *Il2rg* null mutation	Enhanced	Malignant	[57]
NSG	*NOD background (Sirpa*^*Nod*^*)*,* Prkdc*^*scid*^*mutation*, *Il2rg* null mutation	Optimal	Malignant, Spontaneous	[13, 55, 58]
NRG	*NOD background (Sirpa*^*Nod*^*)*,* Rag1 or Rag2 mutation*	Optimal	Malignant	[59]

#### Matching strains to research objectives

CDX models used for routine tumor growth studies and preliminary drug screening are commonly performed in nude mice. Their low cost and ease of handling make them practical for large experiments [60, 61]. PDX and PDOX studies generally require NOD-SCID, NOG, NSG, or NRG recipients because successful engraftment depends on suppression of both adaptive and innate immunity [12, 62, 63]. Among these strains, NSG mice are frequently preferred because of their high engraftment efficiency [12, 62].

Metastasis studies often utilize NOG, NSG, or NRG mice [63]. Reduced NK-cell activity permits survival of circulating tumor cells and facilitates dissemination to distant organs. NSG mice are especially valuable for spontaneous metastasis models because metastatic patterns frequently resemble those observed in patients [13].

Research focused on the tumor microenvironment frequently incorporates co-transplantation of cancer-associated fibroblasts [64], endothelial cells (ECFCs) [12], or immune cells [65]. These complex models are generally established in NOG, NSG, or NRG mice. Humanized models are preferred when evaluation of immunotherapies requires functional human immune responses.

#### Practical considerations affecting strain selection

**Fig. 1:**
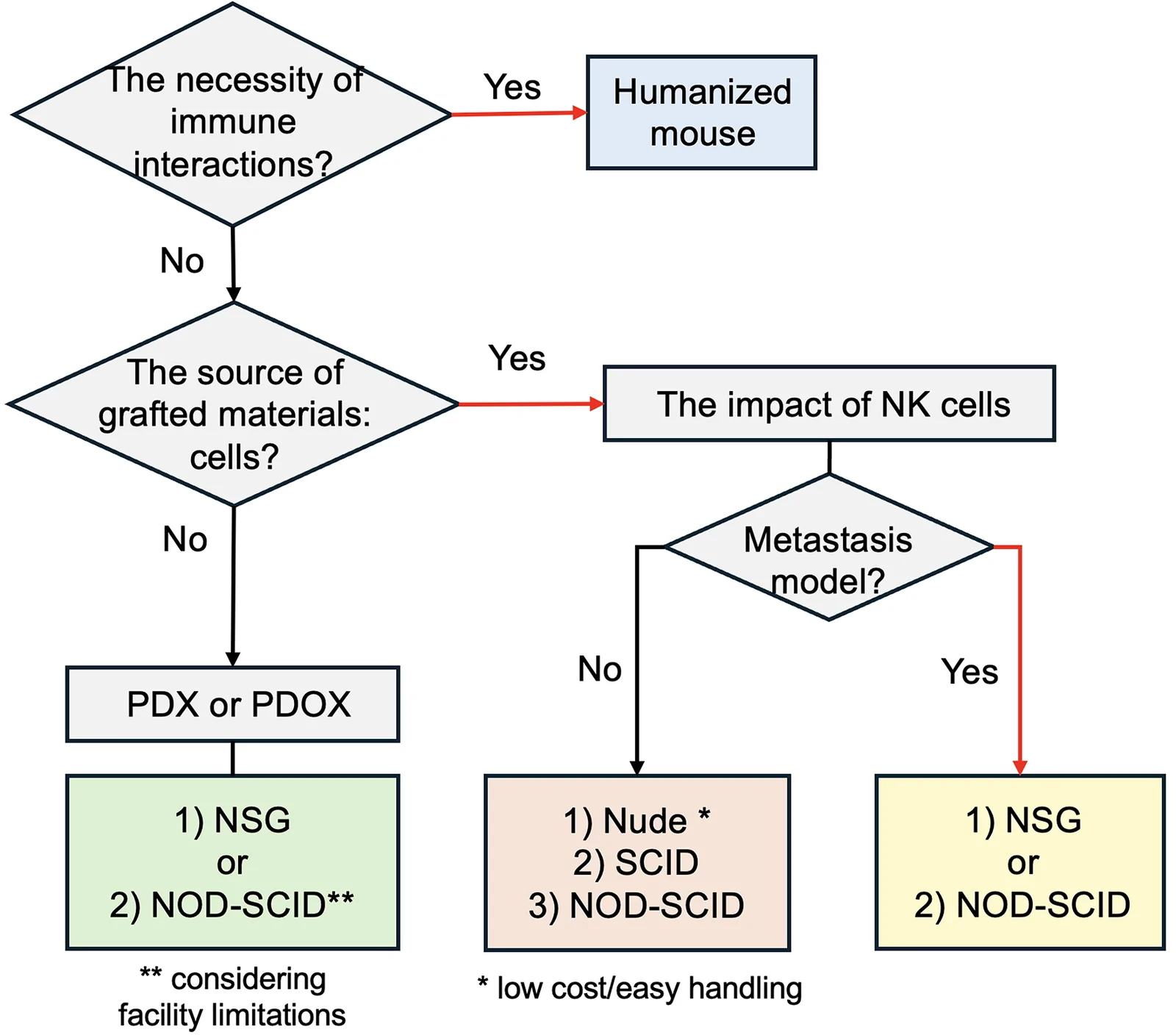
Decision tree for mouse strain selection in xenograft models. A decision tree illustrating the selection of mouse strains for xenograft experiments based on study objectives and immunological requirements. The selection process is guided by key determinants, including the necessity of immune interactions (particularly T-cell involvement), the source of grafted material (CDX versus PDX or PDOX), and the impact of NK cells, on engraftment efficiency. Nude mice, which lack functional T cells but retain B and NK cells, are suitable for basic CDX studies with moderate engraftment demands. SCID and NOD-SCID mice provide progressively greater immunodeficiency, improving xenograft take rates but with limitations related to immune leakiness and lymphoma incidence. NSG mice, deficient in T, B, and NK cells, offer the highest engraftment efficiency and are preferred for challenging CDX, PDX, orthotopic, metastatic, and humanized xenograft models requiring maximal suppression of host immune responses

Beyond biological requirements, practical considerations influence strain selection. Highly immunodeficient and humanized mice require specialized housing conditions and are substantially more expensive than nude or SCID mice. Investigators must therefore balance scientific objectives with available resources (Fig. 1). Animal age is another important variable because immune leakage may occur in some strains over time. Experimental reproducibility can be improved by using age-matched cohorts and carefully monitoring immune status. Imaging requirements should also be considered. Bioluminescent imaging can be performed in all strains. However, hairless nude mice offer advantages for fluorescence-based imaging because fur generates substantial background autofluorescence [66].

## Conclusions

**Fig. 2:**
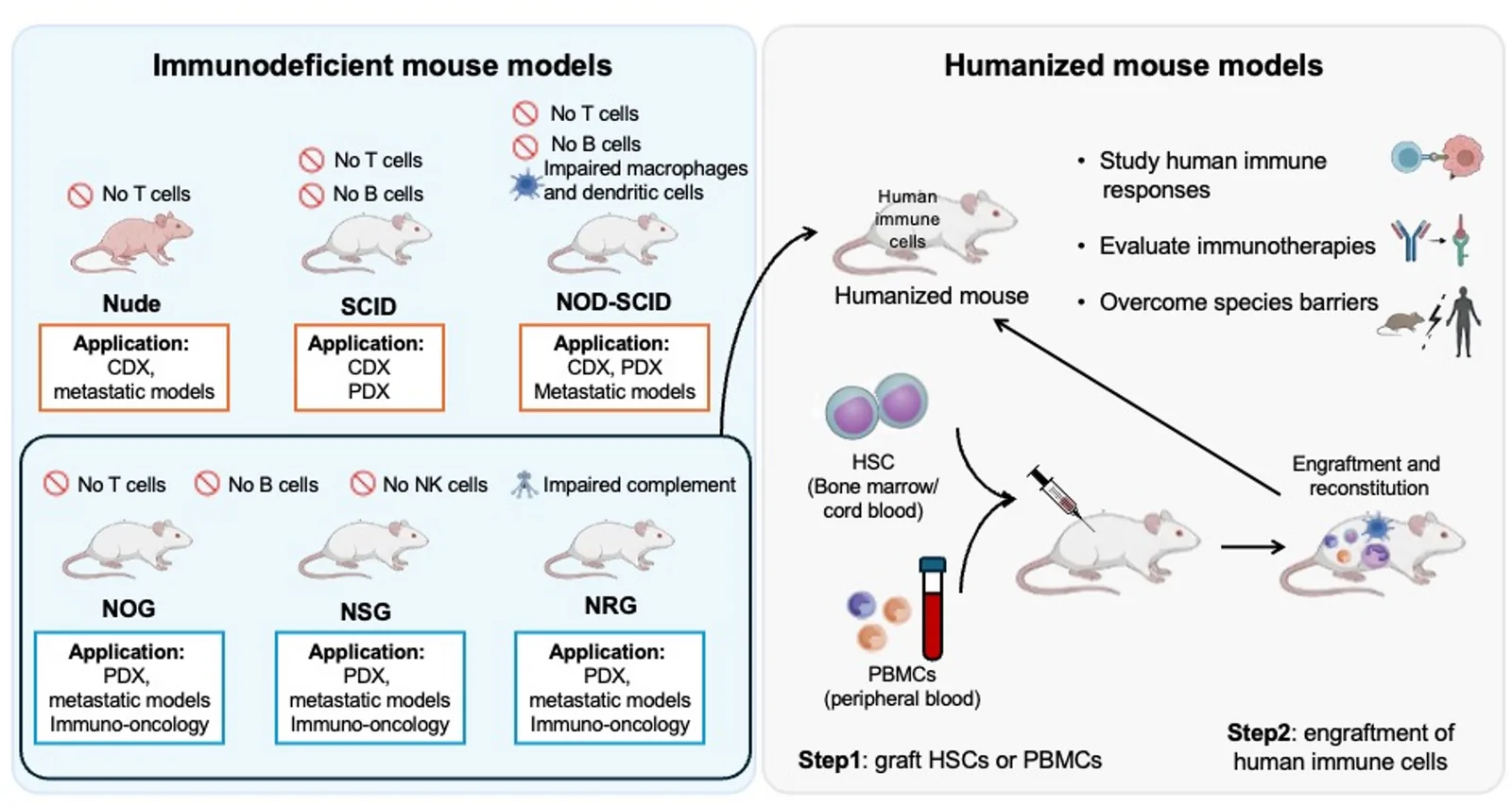
Overview of immunodeficient and humanized mouse models for preclinical cancer research. Immunodeficient mouse strains are essential tools for xenograft and immune-oncology research. Nude, SCID, NOD-SCID, NOG, NSG, and NRG models are categorized by their specific immune deficits and research applications, such as CDX, PDX, and immune-oncology. Humanized models are generated by engrafting human HSCs or PBMCs onto these recipients. These models enable the study of human-specific immune responses, the evaluation of immunotherapies, and the preclinical assessment of human cancer treatments by overcoming species barriers

Xenograft mouse models remain indispensable tools for cancer biology, drug development, metastasis research, and immuno-oncology (Fig. 2). Progressive advances in immunodeficient strains have dramatically improved the ability to engraft human tissues and reproduce clinically relevant tumor behavior. Nude, SCID, NOD-SCID, NOG, NSG, NRG, and humanized mice each provide distinct advantages and limitations. Consequently, no single model is universally optimal. Careful consideration of immune status, research objectives, metastasis requirements, human immune reconstitution, technical feasibility, and cost is essential for selecting the most appropriate model and maximizing experimental success.

## Data Availability

All data generated or analyzed during this study are included in this published article.

## Abbreviations

BLT model: Bone marrow-liver-thymus model
CAF: Cancer associated fibroblasts
CAR-T: Chimeric antigen receptor-T
CDX: Cancer cell line-derived xenograft
ECFC: Endothelial colony-forming cell
GvHD: Graft-versus-host disease
HSC: Human hematopoietic stem cell
NK cell: Natural killer cell
NOD: Non-obese diabetic
NOG: NOD.Cg-Prkdc^scid^ Il2rg^tm1Sug^/Jic, NOD/Shi-scid, IL-2Rγ^null^
NSG: NOD.Cg-Prkdc^scid^ Il2rg^tm1Wjl^/SzJ, NOD/LtSz-scid, IL-2rγ^null^
NRG: NOD.Cg-Rag1^tm1Mom^ Il2rg^tm1Wjl^/SzJ, NOD.Cg-Rag2^tm1Fwa^ Il2rg^tm1Sug^/Rj
PBMC: Peripheral blood mononuclear cell
PDOX: Patient-derived organoid xenograft
PDX: Patient-derived xenograft
SCID: Severe combined immunodeficient
